# Prevalence of sarcopenia in the world: a systematic review and meta- analysis of general population studies

**DOI:** 10.1186/s40200-017-0302-x

**Published:** 2017-05-16

**Authors:** Gita Shafiee, Abbasali Keshtkar, Akbar Soltani, Zeinab Ahadi, Bagher Larijani, Ramin Heshmat

**Affiliations:** 1 Chronic Diseases Research Center, Endocrinology and Metabolism Population Sciences Institute, Tehran University of Medical Sciences, Tehran, Iran; 2 Department of Health Sciences Education Development, School of Public Health, Tehran University of Medical Sciences, Tehran, Iran; 3 Endocrinology and Metabolism Research Center, Endocrinology and Metabolism Clinical Sciences Institute, Tehran University of Medical Sciences, Tehran, Iran; 4 Endocrinology and Metabolism Research Center (EMRC), Dr Shariati Hospital, North Karegar St, Tehran, 14114 Iran

**Keywords:** Sarcopenia, Prevalence, General population, Systematic review

## Abstract

**Background:**

Sarcopenia, an age-related decline in muscle mass and function, is one of the most important health problems in elderly with a high rate of adverse outcomes. However, several studies have investigated the prevalence of sarcopenia in the world, the results have been inconsistent. The current systematic review and meta- analysis study was conducted to estimate the overall prevalence of sarcopenia in both genders in different regions of the world.

**Methods:**

Electronic databases, including MEDLINE (via PubMed), SCOPUS and Web of Science were searched between January 2009 and December 2016. The population- based studies that reported the prevalence of sarcopenia in healthy adults aged ≥ 60 years using the European Working Group on Sarcopenia in Older People (EWGSOP), the International Working Group on Sarcopenia (IWGS) and Asian Working Group for Sarcopenia (AWGS) definitions, were selected. According to these consensual definitions, sarcopenia was defined by presence of low muscle mass (adjusted appendicular muscle mass for height) and muscle strength (handgrip strength) or physical performance (the usual gait speed).

The random effect model was used for estimation the prevalence of sarcopenia. The sex-specific prevalence of sarcopenia and 95% confidence interval (CI) were calculated using the Binomial Exact Method. Heterogeneity was assessed by subgroup analysis.

**Results:**

Thirty- five articles met our inclusion criteria, with a total of 58404 individuals. The overall estimates of prevalence was 10% (95% CI: 8-12%) in men and 10% (95% CI: 8-13%) in women, respectively. The prevalence was higher among non- Asian than Asian individuals in both genders especially, when the Bio-electrical Impedance Analysis (BIA) was used to measure muscle mass (19% vs 10% in men; 20% vs 11% in women).

**Conclusion:**

Despite the differences encountered between the studies, regarding diagnostic tools used to measure of muscle mass and different regions of the world for estimating parameters of sarcopenia, present systematic review revealed that a substantial proportion of the old people has sarcopenia, even in healthy populations. However, sarcopenia is as a consequence of the aging progress, early diagnosis can prevent some adverse outcomes.

## Background

Sarcopenia is an age-related disease described by a progressive loss of muscle mass and function [1]. In addition, sarcopenia is a major clinical problem in public health of older people; with some adverse outcomes such as disability, poor quality of life, and increased risk of death [2–4]. The prevalence of sarcopenia is rising, which is as a result of population aging all over the world. Characteristics of the studied population (such as age, sex, race and differences in body composition in ethnic groups) and the methodology used to assess parameters of sarcopenia cause a wide variation in the rate of this disease [5, 6].

Researches in Europe and Asia have developed the consensus sets of definition and diagnostics criteria for sarcopenia based on its parameters. In 2010, a practical clinical definition was reported by the European Working Group on Sarcopenia in Older People (EWGSOP) [7]. The similar approaches for definition of sarcopenia were taken by the International Working Group on Sarcopenia (IWGS) [8] and Asian Working Group for Sarcopenia (AWGS) [9]. According to these consensual definitions, sarcopenia is defined by presence of low whole body or appendicular skeletal muscle mass in combination with poor muscle function.

There are some different methods used to define cut-off points for muscle mass and other parameters of sarcopenia. Although, the EWGSOP and the AWGS suggested cut-off values for muscle mass and function, the practice of young adult from the same population under study to determine these values is recommended. Hereof, researchers have shown significant differences and bias in the prevalence of Sarcopenia when different cut points of the distinct populations used instead of the original sex- specific group of subjects [10, 11].

Therefore, the use of different measurement tools, cut- off points and definitions may lead to different prevalence of sarcopenia and the results may be difficult to interpret. This may have important consequences on clinical researches and development of preventive and therapeutic strategies. Therefore, the European and Asian working groups recommended developing the systematic reviews of some aspects of sarcopenia. In this regard, we performed this study, aiming to systematically review the finding of all available studies in this issue and estimate the prevalence of sarcopenia using definitions as proposed by the EWGSOP and the AWGS; and also to assess potential sources of heterogeneity.

## Methods

### Search strategy

The search was carried out in three electronic databases, MEDLINE (via PubMed), SCOPUS and Web of Science between January 2009 and December 2016. The pre-defined search terms were: “sarcopenia” or “muscle mass” and “aging” or “older people” or “elderly” and “prevalence” or “epidemiology” or “frequency” or ‘incidence”. The list of references of articles was also reviewed for any additional papers. Furthermore, specialized journals and textbooks that were related to the topic were reviewed to gather data on sarcopenia prevalence. In addition, gray literatures, such as reports and conference presentations, were considered using the Google search engine to further ensure that pertinent articles were not missed. The reference lists of included studies were screened as well. Search was not limited by language.

### Study selection

We included only studies that had enrolled community-dwelling participants aged 60 years and older among the normal population and population base approach sampling studies. Studies that prevalence of sarcopenia had been assessed according to the EWGSOP, AWGS or IWGS definitions of sarcopenia, i.e. based on muscle mass (adjusted appendicular muscle mass for height) and muscle strength (handgrip strength) or physical performance (the usual gait speed), were included. They were excluded if they only used muscle mass to define sarcopenia, and studies that were from clinical/hospital or care setting. Also, studies that were not published as full report like conferences abstract and letters to editors, case-report studies were excluded.

Research papers were selected based on titles and abstracts. The full texts of all selected publications were assessed for pertinence. If the full texts of the papers were not available, they were obtained through correspondence with the authors. The process initially performed by two investigators. A third investigator adjudicated discrepancies between the two reviewers.

### Data extraction and quality evaluation

The methodological quality of the eligible articles was assessed using a modified STROBE checklist [12]. Two reviewers evaluated each article’s quality based on the checklist, and if a consensus was not reached, a third reviewer reassessed the manuscript. We extracted from the articles the study characteristics (e.g. publication year, country of data collection), characteristics of target population (e.g. age, gender and sample size), assessment method used for each parameter (muscle mass, muscle strength, and muscle performance), cut-off values of each parameter, and sarcopenia prevalence.

### Statistical analysis

Data were presented as numbers and proportions. The random effect model was used for combining the prevalence of sarcopenia. The sex-specific prevalence of sarcopenia and 95% confidence interval (CI) were calculated using the Binomial Exact Method. Heterogeneity was assessed by subgroup analysis. The heterogeneity of studies greater than 50% indicated severe heterogeneity. The publication bias was assessed by Begg’s test. Given the fact that prevalence does not have a normal distribution, a prevalence index was first calculated using the logit of prevalence and the standard error of logit prevalence, and then tests were done. All meta-analysis methods were performed using STATA (Release 12. statistical software. College Station, Texas: STATA Corp LP).

## Results

**Fig. 1:**
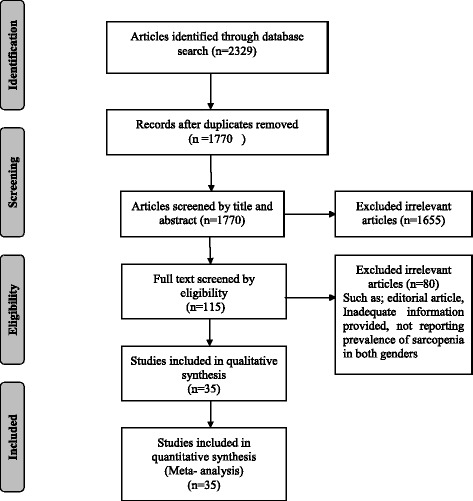
Flow diagram of literature search

The literature searches yielded 2329 articles (including 559 duplicates). According to inclusion criteria, we assessed titles and abstracts and 115 articles were selected. After reviewing the full texts, thirty five studies were eligible for the meta- analysis (Fig. 1).

A total of 58404 individuals, 32642 (55.9%) men and 25762 (44.1%) women; respectively, from the general population were examined through these studies. Twenty one studies were distributed in Asia and there were also 14 studies from non-Asian countries. A total of 18 studies used the Dual Energy X-Ray Absorptiometry (DXA) to assess muscle mass and 16 studies used the Bio-electrical Impedance Analysis (BIA). In a study were used both methods to assess of muscle mass [13]. Characteristics of studies included in the analyses are described in Table 1.

**Table 1:** Characteristics of studies included in the sarcopenia prevalence meta-analysis in healthy ageing adults

Studies	Region (Asia/Non Asia)	Sample (N) Total-Male-Female	Assessment method for muscle mass	Prevalence		
				Total *N* (%)	Male *N* (%)	Female *N* (%)
Htun N.C, et al. 2016 [27]	Asia	1921-976-945	DXA	248 (13.3)	101 (10.34)	147 (16.56)
Borg S.T, et al. 2016 [28]	Non Asia	227-110-117	BIA	53 (23.0)	25 (22.73)	28 (23.93)
Brown J. C, et al. 2016 [29]	Non Asia	4425-1925-2500	BIA	1618 (36.0)	862 (44.8)	756 (30.24
Chan R, et al. 2016 [30]	Asia	3957-1979-1978	DXA	290 (7.30)	185 (9.30)	105 (5.30)
Jung H. W, et al. 2016 [31]	Asia	382-167-215	BIA	105 (27.80)	46 (28.10)	59 (27.44)
Han D. S, et al. 2016 [32]	Asia	878-402-476	BIA	29 (3.3)	27 (6.7)	2 (0.4)
Spira D, et al. 2016 [33]	Non Asia	1405-622-783	DXA	58 (4.10)	40 (6.40)	18 (2.30)
Bianchi L, et al. 2015 [34]	Non Asia	538-250-288	BIA	55 (10.20)	19 (7.60)	36 (12.50)
Han P, et al. 2016 [35]	Asia	657-216-441	DXA	64 (9.70)	21 (9.7)	43 (9.8)
Huang C.Y, et al. 2016 [36]	Asia	731-386-345	DXA	50 (6.80)	36 (9.30)	14 (4.10)
Siva Neto L.S, et al. 2016 [37]	Non Asia	70-31-39	DXA	7 (10.0)	5 (16.10)	2 (5.10)
Han P, et al. 2016 [38]	Asia	1069-470-606	BIA	99 (9.30)	30 (6.40)	69 (11.50)
Velazquez-Alva M.C, et al. 2015 [39]	Non Asia	-,-,137	DXA	-	-	20 (14.60)
Wang Y. J, et al. 2015 [40]	Asia	316-164-152	DXA	94 (29.75)	43 (26.20)	51 (33.55)
Pereira F. B, et al. 2015 [41]	Non Asia	-,198,-	DXA	-	20 (10.10)	-
Cawthon P.M, et al. 2015 [42]	Non Asia	-,5934,-	DXA	-	277 (4.70)	-
Meng N. H, et al. 2015 [43]	Asia	771-412-359	DXA	44 (5.70)	35 (8.40)	9 (2.60)
Wen X, et al. 2015 [44] ^a^	Asia	286-136-150	DXA			
1				17 (5.90)	10 (7.40)	7 (4.70)
2				9 (3.14)	8 (5.90)	1 (0.70)
3				1 (0.35)	1 (0.80)	0 (0.00)
Bischoff-Ferrari H.A, et al. 2015 [10] ^a^	Non Asia	445-199-246	DXA			
1				31 (7.10)	13 (6.60)	18 (7.40)
2				22 (5.00)	7 (3.60)	15 (6.20)
3				12 (2.70)	6 (3.10)	6 (2.50)
Nishiguchi S, et al. 2015 [45]	Asia	-,-,273	BIA	-	-	22 (8.06)
Beaudart C, et al. 2014 [46] ^a^	Non Asia	400-157-243	DXA			
1				61 (15.20)	23 (14.65)	38 (15.63)
2				72 (18.00)	23 (14.65)	49 (20.16)
Yoshida D, et al. 2014 [47]	Asia	4811-2343-2468	BIA	360 (7.50)	192 (8.20)	168 (6.80)
Tanimoto Y, et al. 2014 [48]	Asia	1110-372-738	BIA	160 (14.41)	50 (13.40)	110 (14.90)
Wu C.H, et al. 2014 [49]	Asia	549-285-264	BIA	39 (7.10)	11 (3.86)	28 (10.61)
Yu R, et al. 2014 [50]	Asia	4000-2000-2000	DXA	293 (7.32)	187 (9.35)	106 (5.30)
Akune T, et al. 2014 [51]	Asia	1000-349-651	BIA	129 (12.90)	48 (13.80)	81 (12.40)
Dam T. T, et al. 2014 [52] ^a^	Non Asia	10,063-7113-2950	DXA			
1				710 (7.0)	362 (5.10)	348 (11.80)
2				768 (7.63)	376 (5.30)	392 (13.30)
Wu I.C.et al. 2014 [11] ^a^	Asia	2867-1431-1436	BIA			
1				50 (1.74)	33 (2.30)	17 (1.18)
2				105 (3.70)	55 (3.84)	50 (3.50)
Ishii S, et al. 2014 [53]	Asia	1971-977-994	BIA	359 (18.21)	139 (14.20)	220 (22.10)
Pagotto V, et al. 2014 [13] ^a^	Non Asia	132-52-81				
1			DXA	17 (13.00)	8 (15.40)	9 (11.40)
2			DXA	22 (16.80)	8 (15.40)	14 (17.28)
3			DXA	48 (36.60)	12 (23.10)	36 (44.44)
4			BIA	40 (30.50)	13 (25.00)	27 (33.33)
5			BIA	23 (17.60)	8 (15.40)	15 (19.0)
Yu S, et al. 2014 [54] ^a^	Non Asia	986-611-375	DXA			
1				16 (1.6)	15 (2.5)	1 (0.3)
2				73 (7.4)	38 (6.2)	35 (9.3)
Volpato S, et al. 2014 [55]	Non Asia	730-345-385	BIA	55 (7.50)	19 (5.51)	36 (9.35)
LeeW.J, et al. 2013 [56] ^a^	Asia	386-223-163	DXA			
1				30 (7.80)	24 (10.80)	6 (3.70)
2				16 (4.10)	13 (5.80)	3 (1.80)
Yamada M, et al. 2013 [57]	Asia	1882-568-1314	BIA	414 (22.0)	124 (21.80)	290 (22.10)
Tanimoto Y. et al. 2012 [58]	Asia	1158-364-794	BIA	126 (10.90)	41 (11.30)	85 (10.70)

*DXA* Dual Energy X-Ray Absorptiometry, *BIA* Bio-electrical Impedance Analysis

^a^ Consists of different methods or definition for estimation of prevalence of sarcopenia

### Meta- analysis

To explore the sources of heterogeneity, eight subgroups were analyzed. These groups were gender, region of the study, and method of muscle mass measurement. Then, we assessed region and method in each gender, separately. The overall estimates of prevalence was 10% (95% CI: 8–12%) in men and 10% (95% CI: 813%) in women, respectively. Substantial heterogeneity observed in men (df = 46, I2 = 98.11%, *P* = 0.00) and women (df = 45, I2 = 98.07%, *P* = 0.00). Because the cut-off points of sarcopenia parameters are different in men and women, the prevalence of this disease has been reported in both genders separately. Individuals in non-Asian countries were more likely to be sarcopenic than Asian countries among both genders (11% vs 10% in men, 13% vs 9% in women) (Data not shown).

**Fig. 2:**
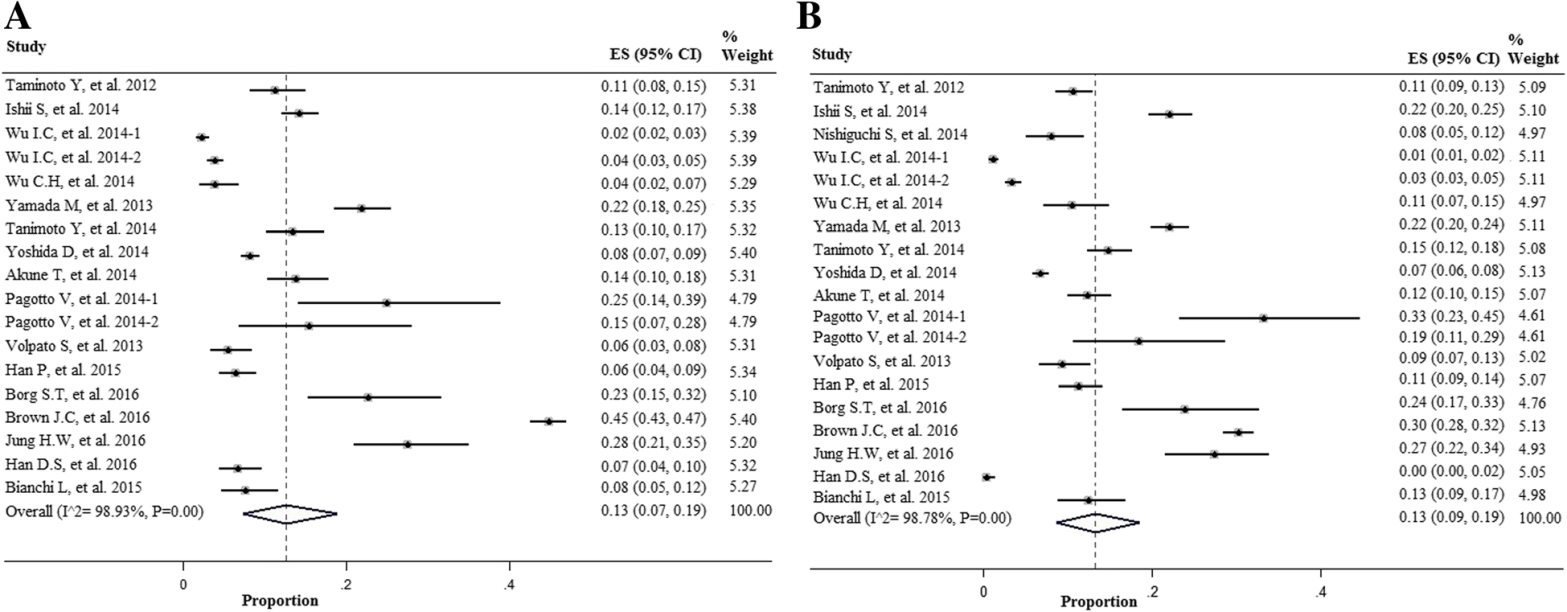
Forest plot of the studies on sarcopenia prevalence by using the Bio-electrical Impedance Analysis (BIA) in both genders; **a** Men; **b** Women

**Fig. 3:**
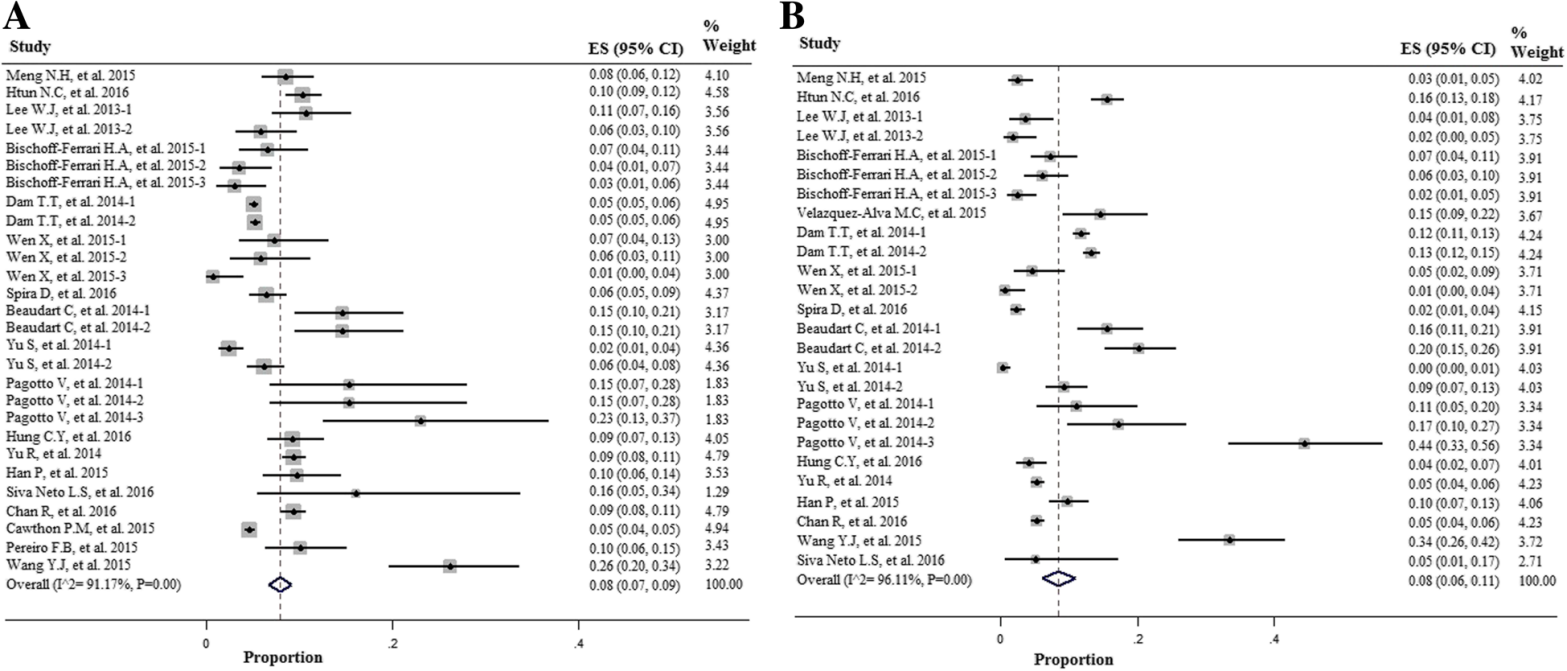
Forest plot of the studies on sarcopenia prevalence by using the Dual Energy X-Ray Absorptiometry (DXA) in both genders; **a** Men; **b** Women

As shown in Fig. 2, BIA had influence on the prevalence of sarcopenia in men and women, respectively [13% (7–19%) in men; 13% (9-19%) in women]. When the method DXA was used to measure muscle mass, the prevalence of sarcopenia was lower in both genders [8% (7–9%) in men; 8% (6–11%) in women] (Fig. 3).

**Fig. 4:**
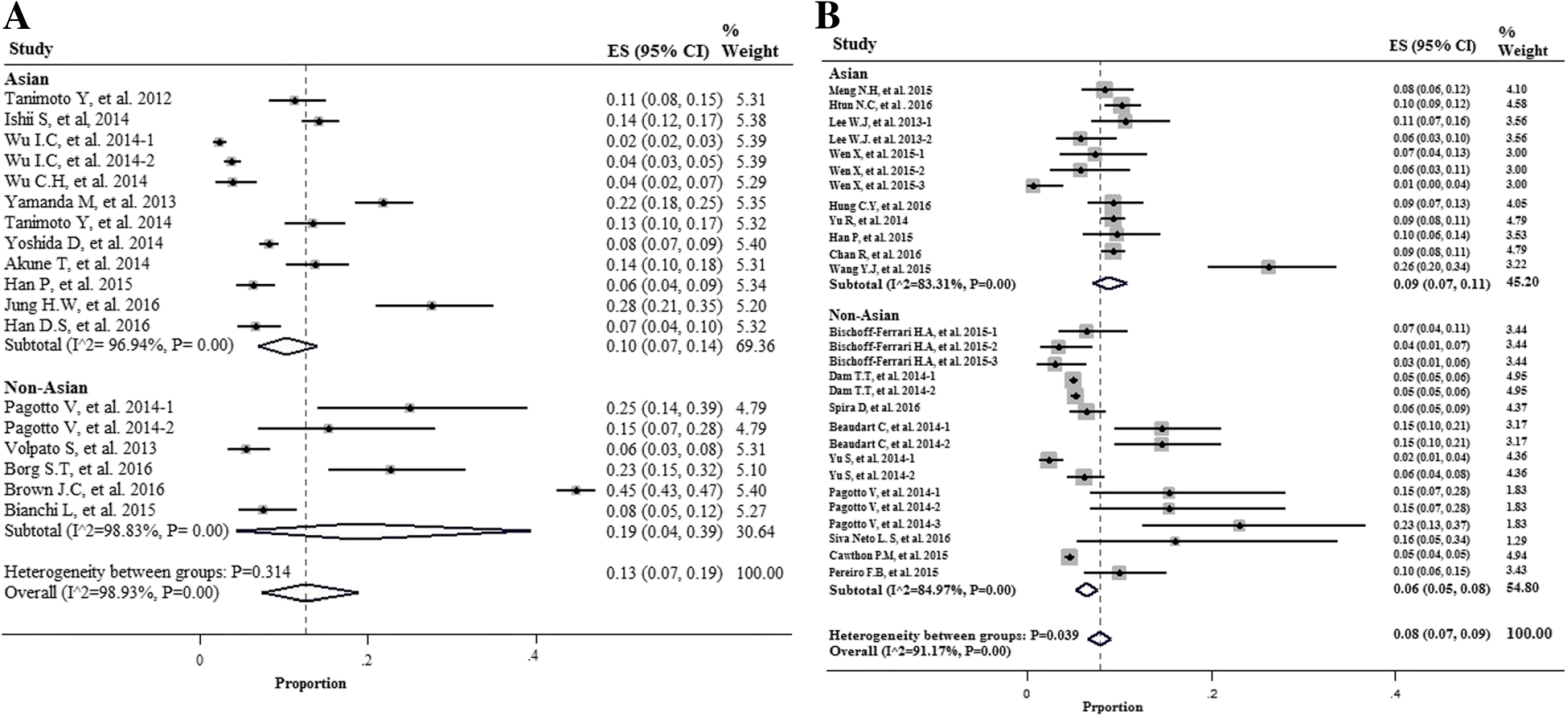
Forest plot of the studies on sarcopenia prevalence by using the Bio-electrical Impedance Analysis (BIA) **a** and the Dual Energy X-Ray Absorptiometry (DXA) **b** according to region of study (Asia & Non- Asian) in Men

In the non-Asian subgroup, the prevalence of sarcopenia among men was higher than in the Asian population when the method BIA was used to measure muscle mass (19 vs 10%). However, in the studies that DXA was used, the prevalence of sarcopenia was 9% in Asian men and 6% in non-Asian men (Fig. 4).

**Fig. 5:**
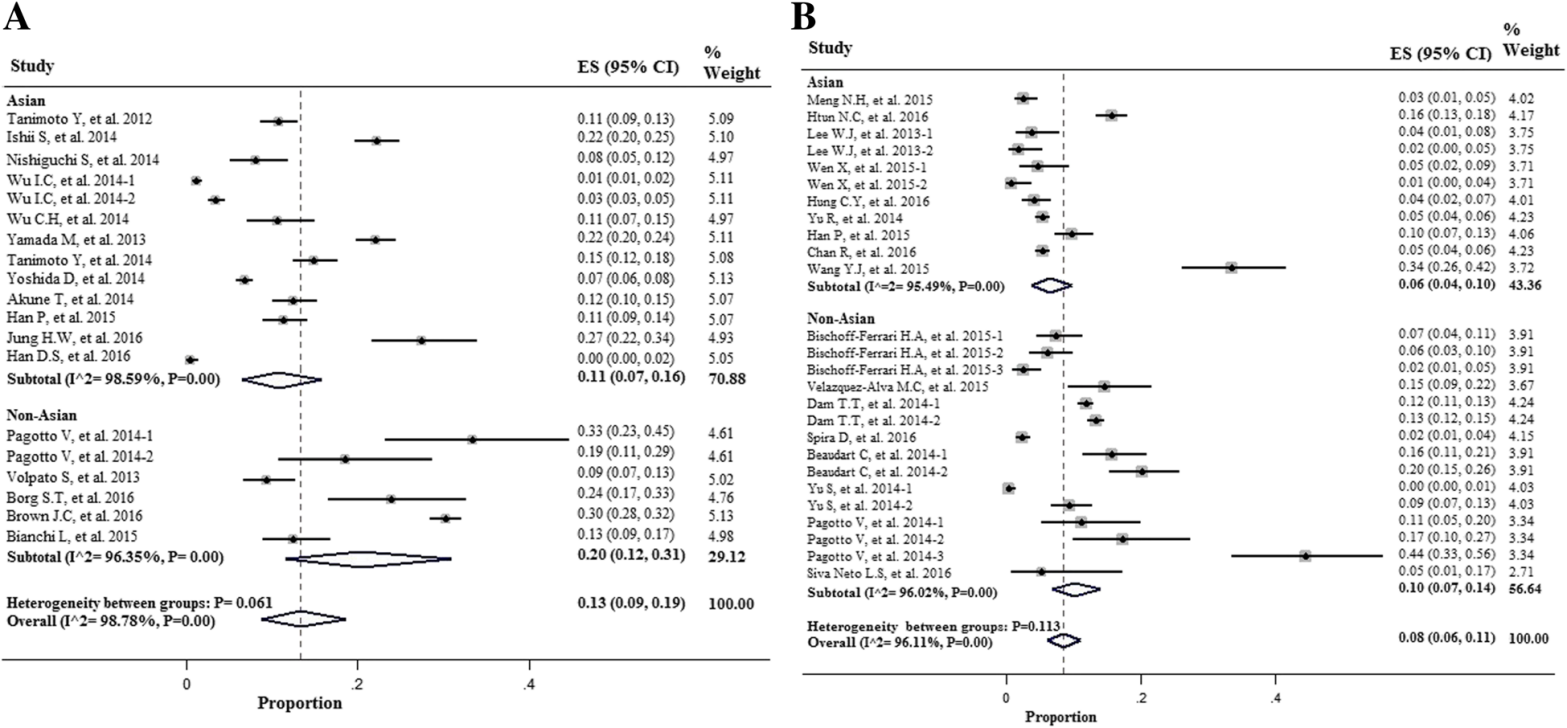
Forest plot of the studies on sarcopenia prevalence by using the Bio-electrical Impedance Analysis (BIA) **a** and the Dual Energy X-Ray Absorptiometry (DXA) **b** according to region of study (Asia & Non- Asian) in Women

Women in non-Asian countries were more likely to be sarcopenic than their counterparts in Asia in both tools for measurement of muscle mass [20 vs 11% with BIA method; 10 vs 6% with DXA method) (Fig. 5).

According to Begg’s test the value for Z equaled 1.79 (*p* = 0.08) in men and 0.31 (*p* = 0.76) in women, that showed no evidence of substantial publication bias.

## Discussion

With increase in the world’s older population, sarcopenia is becoming a serious global public health problem. Based on the results of this meta- analysis, despite the high heterogeneity of prevalence estimates across primary studies, the overall prevalence of sarcopenia was 10%. In the non-Asian countries, the prevalence of sarcopenia was more likely than in the Asian individuals. BIA had influence on the prevalence of sarcopenia in both genders. Considering that sarcopenia has developed a more important issue in elderly, we presumed that an estimate of prevalence in both genders by meta-analysis would be necessary for further strategies of prevention or treatment of sarcopenia.

However, the prevalence of sarcopenia was similar in both genders in this systematic review; the differences have been shown in previous studies. Gender and age differences in body composition are well known. Regarding the association between sex and sarcopenia, results are inconsistent. Some studies [5, 14] reported higher relative reduction of muscle mass in men than in women and therefore, the highest prevalence of sarcopenia (50%) was found among men older than 80 years while only 43.8% of the women of the same age group corresponded to the definitions of sarcopenia [1, 15, 16]. In contrast, other studies yielded high rates of sarcopenia among women younger than 80 years [16]. Various endogenous and exogenous factors influence on prevalence of sarcopenia. Furthermore hormonal changes which enhance the decrease of muscle mass occur more slowly in men than in women. After menopausal transition, the concentrations of sex steroids, both estrogens and androgens, decrease dramatically [17]. In men, the decline of sex steroids is much slower than in women [18]. This advanced decrease of anabolic acting androgens in women may be one important factor in explaining the significantly higher prevalence of sarcopenia among women ageing between 60 and 70 years. After the eighth decade of life, testosterone concentrations in human males decline rapidly which may contribute to the decrease in lean body mass and the increase in sarcopenia.

In present review, we just have sub grouped the studies according to gender, and the studies that had enrolled only participants in the age group above 70 years, were excluded. Also, a pooled analysis of different age groups was not conducted due to inadequate data in each subgroup. Therefore, it seems similar prevalence of sarcopenia in both genders is due to these items.

In relation to the type of elderly population, the prevalence of sarcopenia was higher in elderly people in the rehabilitation units, followed by nursing homes and hospitalized in previous studies [19–21]. Therefore, we excluded these studies that may overestimate its prevalence.

Within our expectation, some variations in estimates of prevalence of sarcopenia were found in sub groups. This variability was not explained by region, and tool of muscle mass measurement. However, some of these variables did change the estimated prevalence of sarcopenia in both genders. The pooled prevalence was higher among non-Asian individuals than in the Asian population in both genders (11 vs 10% in men, 12 vs 9% in women).

These results can be attributed to racial characteristics, body size, cultural background, dietary regimes, and life quality of the elderly between the Asian and non-Asian individuals in different countries. Also, the cut-off points for the Asian populations [9] are lower than for the non-Asian individuals [7] in both genders, with young people of the same ethnic group as reference. In addition, the mean appendicular muscle mass of young Asians was about 15% lower than that of non- Asians even after height adjustments [1, 22]. Therefore, low muscle mass in young Asians will effect in lower prevalence of sarcopenia in the old people. Furthermore, sarcopenia may be less prevalent in Asians due to differences in lifestyle such as a better dietary aspect and higher levels of activity than the Western populations, which act as protective factors against sarcopenia [23].

Another important factor to estimate prevalence of sarcopenia is tools that use to evaluate muscle mass and diagnose sarcopenia. However, the EWGSOP and other groups do not recommend the use of specific tools to measure muscle mass and other parameters of sarcopenia, but they suggest DXA and BIA to assess muscle mass [7]. The use of different diagnostic tools may lead to different prevalence of sarcopenia and may therefore have important consequences on clinical researches and development of therapeutic strategies. BIA is known to underestimate fat mass and overestimate muscle mass [24]. Previous studies found that the BIA-based prevalence of sarcopenia was higher than the DXA- based approach [25, 26]. Also, we found important differences of measured prevalence of sarcopenia whether BIA or DXA. When the BIA was used, the pooled prevalence in both genders was observed to be 13% in men and women. This positive correlation implies an even higher prevalence of sarcopenia among the region. These results suggest that the prevalence of sarcopenia should be tool-based approach dependent.

The limitations of the present review need to be considered. First, not all studies presented data according to age group; therefore, we could not estimate the prevalence for each age subgroup. Second, grouping according to different cut-off points for parameters of sarcopenia and different definitions were led to reader’s confusion. So, analyses were based on sex, region and tool of muscle mass measurement. Third, characteristics of residents (e.g. rural or urban) could not be assessed due to insufficient data from the majority of publications.

One of the strength of the present systematic review and meta- analysis was the first study that only included studies that were conducted in the general population. Other strength of this study was that only high quality articles were included. Most of the excluded articles either had a low quality based on STROBE quality assessment forms and therefore did not present reliable data, or lacked information required for the review, such as adequate descriptive information about the population of their study, or a separate sarcopenia prevalence in different sex, which can make it difficult to use in a meta-analysis.

## Conclusion

Despite the differences encountered between the studies, regarding diagnostic tools used to measure of muscle mass and different regions of the world for estimating parameters of sarcopenia, present systematic review revealed that a substantial proportion of the old people has sarcopenia, even in healthy populations. However, sarcopenia is as a consequence of the aging progress, early diagnosis of those at risk can prevent some adverse outcomes. Therefore, future researches are needed to find a consensus regarding the tools that must be used in the context of diagnosing and screening of sarcopenia. Therefore, it is necessary that the simple and valid methods for diagnosing and screening of sarcopenia used in clinical practice.

## Abbreviations

AWGS: Asian Working Group for Sarcopenia
BIA: Bio-electrical impedance analysis
DXA: Dual Energy X-Ray Absorptiometry
EWGSOP: European Working Group on Sarcopenia in Older People
IWGS: International Working Group on Sarcopenia
STROBE: Strengthening the Reporting of Observational studies in Epidemiology
